# HEG1 as a novel potential biomarker for the prognosis of lung adenocarcinoma

**DOI:** 10.1002/cam4.5081

**Published:** 2022-08-10

**Authors:** Xin Zou, Yue Zhang, Ning Wang, Jie Shi, Qinghai Li, Wanming Hao, Wenjing Zhu, Wei Han

**Affiliations:** ^1^ Department of Pathology, Qingdao Municipal Hospital Dalian Medical University Qingdao China; ^2^ Respiratory Disease Key Laboratory of Qingdao Qingdao Municipal Hospital Qingdao China; ^3^ Department of Respiratory Jilin Provincial People's Hospital Jilin China; ^4^ Department of Pulmonary and Critical Care Medicine, Qingdao Municipal Hospital, School of Medicine Qingdao University Qingdao China; ^5^ NMPA Key Laboratory for Quality Research and Evaluation of Traditional Marine Chinese Medicine Qingdao China; ^6^ Clinical Research Center, Qingdao Municipal Hospital Qingdao University Qingdao China

**Keywords:** biomarker, HEG1, lung adenocarcinoma, prognosis

## Abstract

**Background:**

Heart development protein with EGF‐like domains 1 (HEG1), generally related to angiogenesis and embryonic development, was reported to participate in the occurrence and progression of some tumors recently. However, the role of HEG1 in lung adenocarcinoma (LUAD) is unclear.

**Patients and Methods:**

To explore the effect of HEG1 on LUAD, GEPIA platform and UALCAN database, as well as Kaplan–Meier plotter were adopted to analyze the association of HEG1 with clinicopathological characteristics and survival outcomes for LUAD firstly. And then the HEG1 in LUAD tissues, blood and cell lines were detected by qRT‐PCR, western blot, immunofluorescence, immunohistochemistry, and ELISA. Gene set enrichment analysis (GSEA) was conducted to identify pathways that might be affected by HEG1 in LUAD.

**Results:**

In this study, HEG1 in lung tissues and cell lines of LUAD were significantly downregulated compared to benign pulmonary disease tissues and alveolar epithelial cells (*p* < 0.05). Moreover, compared with other groups, patients with advanced tumor stage had lower HEG1 mRNA expression levels (*p* = 0.025), which were negatively correlated with Ki67 index in tumor tissues (*r* = −0.427, *p* = 0.033). On the other hand, the LUAD patients with lower HEG1 had shorter overall survival (OS) (HR = 0.51, 95% CI: 0.40–0.65, *p* < 0.001) according to Kaplan–Meier plotter. In addition, HEG1 in serum of LUAD patients was negatively associated with CEA (*r* = −0.636, *p* < 0.001). GSEA showed that HEG1 was enriched in various metabolic‐related pathways, including glucose metabolism, lipid metabolism, and nucleotide metabolism signaling.

**Conclusions:**

HEG1 was downregulated in LUAD patients and associated with poor prognosis, which indicating HEG1 may serve as a potential biomarker for diagnosis and prognosis of LUAD.

## Graphical abstract

HEG1 as a novel potential biomarker for the prognosis of lung adenocarcinoma. The patients with low HEG1 expression may have a relatively malignant procession.

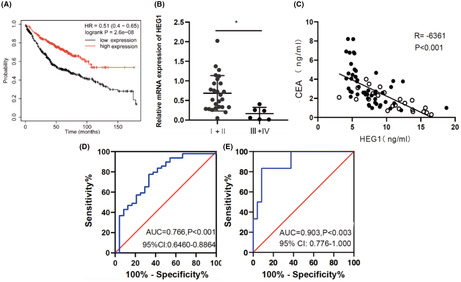

## 1 INTRODUCTION

Lung cancer is one of the most common malignant tumors. It is estimated that there will be 2.2 million new cases and 1.8 million deaths in 2020 according to the Global Cancer Statistics.^1^ Lung adenocarcinoma (LUAD), as the predominant histological subtype of lung cancer, causes over 1 million deaths worldwide annually, resulting from the lack of typical symptoms in the early stage.^2^, ^3^ Recently, a series of somatic mutations of driver genes, including ALK, KRAS, MET and EGFR, have been found to be related to the treatment and prognosis of LUAD.^4^, ^5^, ^6^ Despite recent advances in the exploration of the molecular mechanism and the development of multidisciplinary comprehensive treatments, the overall 5‐year survival rate of patients of LUAD remains less than 20%.^7^, ^8^ Hence, seeking novel molecular targets that facilitate LUAD initiation and metastasis may provide new insights into the diagnosis and clinical treatment of LUAD.

Heart development protein with EGF‐like domains 1 (HEG1) has been discovered in the study of zebrafish heart in 2003,^9^, ^10^ and been proven to be expressed in various tissues involved in a variety of physiological activities, including angiogenesis, vascular integrity, and embryonic development. It is well‐known that the abnormality in those activities is closely related to tumor development and progression, which indicates that HEG1 might play some critical role in the development of carcinoma.^11^, ^12^, ^13^ In hepatocellular carcinoma, HEG1 has been reported to promote the invasion, metastasis, and epithelial‐mesenchymal transition of HCC by activating Wnt/β‐catenin signaling pathway, so it has been considered as a potential prognostic marker and therapeutic target for HCC.^14^ In addition, recent research has reported that HEG1 is dysregulated in malignant mesothelioma and serves as a stable diagnostic marker for malignant mesothelioma.^13^, ^15^ However, the role of HEG1 in LUAD is unclear.

Herein, this study was conducted to explore the role of HEG1 in LUAD and its underlying mechanism, which might provide new insights into judging the prognosis of LUAD.

## 2 STUDY DESIGN AND METHODS

### 2.1 Patients and lung adenocarcinoma samples

All patients were enrolled from the Department of Respiratory Diseases and Thoracic Surgery before any treatments, including chemotherapy, radiotherapy, or targeted therapy from Qingdao Municipal Hospital; while all healthy volunteers were enrolled from the Health Check‐up Center of Qingdao Municipal Hospital. Lung cancer tissues, paired normal tissues, and lung tissues were obtained from 32 LUAD patients and 12 benign patients, whose clinical data were collected from the electronic medical record system (EMRS). Serum samples were harvested from 59 LUAD patients and 28 healthy volunteers. This study was approved by the ethical committee of Qingdao Municipal Hospital (no. 2019–109) on December 7, 2019.

### 2.2 Cell lines and cell culture

Three cell lines including A549, H1299, and BEAS‐2B were included in our research. Cells were incubated in RPMI 1640 Medium (Gibco Laboratories) supplemented with 10% fetal bovine serum (FBS) (Gibco) and 1% penicillin–streptomycin antibiotic mixture (Gibco) in a 5% CO_2_ incubator at 37°C. All cell lines were authenticated by STR profiling (Procell).

### 2.3 RNA extraction and quantitative real‐time PCR

Total RNA was extracted using TRIzol reagent (Sigma) according to the manufacturer's instructions. Primescript RT reagent Kit (Takara) and TB Green® Premix Ex Taq II (Takara) were adopted to conduct reverse transcription and qRT‐PCR. Primers were obtained from Sangon Biotech, and the sequence of primers as shown in Table 1.

**TABLE 1:** PCR primer sequences used in this study

Gene	Sequences
HEG1	Forward: 5'‐AAGGAACCGAGTGATTGTGG‐3′
	Reverse: 5'‐ACGTGAAGCTGGGCTGTACT‐3′
GAPDH	Forward: 5'‐CAACGTGTCAGTGGTGGACCTG‐3′
	Reverse: 5′‐GTGTCGCTGTTGAAGTCAGAGGAG‐3′

### 2.4 Western blot analysis

Extracted protein was separated by SDS‐PAGE. After being blocked in 5% skimmed milk, the protein‐transferred PVDF membranes (Millipore) were incubated overnight at 4°C in primary antibodies (HEG1, 1:1000 dilution, HPA010952, Sigma; Tubulin,1:1000 dilution, E‐AB‐20070, Elabscience), followed by incubation with second antibodies for 2 h with the dilution of 1:3000. Chemiluminescent and Fluorescent Imaging System was used to detect changes in protein levels.

### 2.5 Immunohistochemical staining

Tissues were fixed with 10% formalin for 24 h. Paraffin sections were dewaxed, dehydrated in gradient. Antigen was repaired with EDTA buffer. Blocking was performed with 1% hydrogen peroxide and goat serum (ZSGB‐BIO). Slides were incubated with primary antibodies (HEG1, 1:100 dilution, HPA010952, Sigma; Ki67, 1:100 dilution, MAB‐0672 MXB; P53,1:200 dilution, MAB‐0674, MXB) overnight at 4°C. Detection was performed with secondary HRP‐conjugated antibodies with DAB as the chromogen. Two independent assessors classified the intensity of immunostaining. The images were documented using a Nikon microscope.

### 2.6 Immunofluorescence

The paraffin sections were dewaxed, dehydrated in gradient. Antigens were repaired with EDTA buffer. Slices were incubated in the primary antibodies (HEG1, 1:100 dilution, HPA010952, Sigma; α‐SMA,1:100 dilution, E‐AB‐34268, Elabscience) overnight at 4°C, and the secondary antibody was incubated at room temperature in the dark. Fluorescent Microscopy is used to detect microscopy and images.

### 2.7 Enzyme‐linked immunosorbent assay

HEG1 concentration in serum was measured according to the instructions of Human HEG1 ELISA kit (Neo Scientific). Absorbance of samples was measured at wavelengths of 450 nm by a microplate reader (Thermo Fisher). The concentration was obtained according to the standard curve provided by the manufacturer.

### 2.8 Bioinformatic analysis

GEPIA platform (http://gepia.cancer‐pku.cn) and UALCAN database (http://ualcan.path.uab.edu/) were used to analyze the expression of HEG1 in LUAD tissues and adjacent normal tissue. Kaplan–Meier plotter (http://kmplot.com/analysis/) was adopted to confirm the effect of HEG1 on the prognosis of LUAD patients. Patients with LUAD were divided into two groups according to the median value of HEG1, and single gene set enrichment analysis (GSEA) was used to confirm the different pathways in the two groups. Gene set permutation was conducted 1000 times for each analysis. Significantly enriched gene sets were those with a normal *p* value of less than 5% and a false discovery rate of less than 25%.

### 2.9 Statistical analysis

SPSS 24.0 software and GraphPad Prism 8.0 were utilized for statistical analysis. The data were expressed as the mean standard deviation at least three times. Student's *t*‐test was used to compare quantitative data between two groups, and one‐way analysis of variance (ANOVA) was used to assess differences among numerous groups; pairwise comparisons were performed using LSD. Fisher's test was used to determine the statistical significance of HEG1 expression and clinicopathological characteristics. The area under the curve (AUC) was calculated using the receiver operating characteristic curve (ROC) to evaluate the diagnostic value of HEG1 for LUAD. The expression level of HEG1 in serum was inverse log transformed as these transformations normalized the skewed distribution of the raw data to meet model assumptions of normality.^16^ Statistical significance was defined as *p* < 0.05.

## 3 RESULTS

### 3.1 Bioinformatic analysis showed that HEG1 dysregulated in lung adenocarcinoma

**FIGURE 1:**
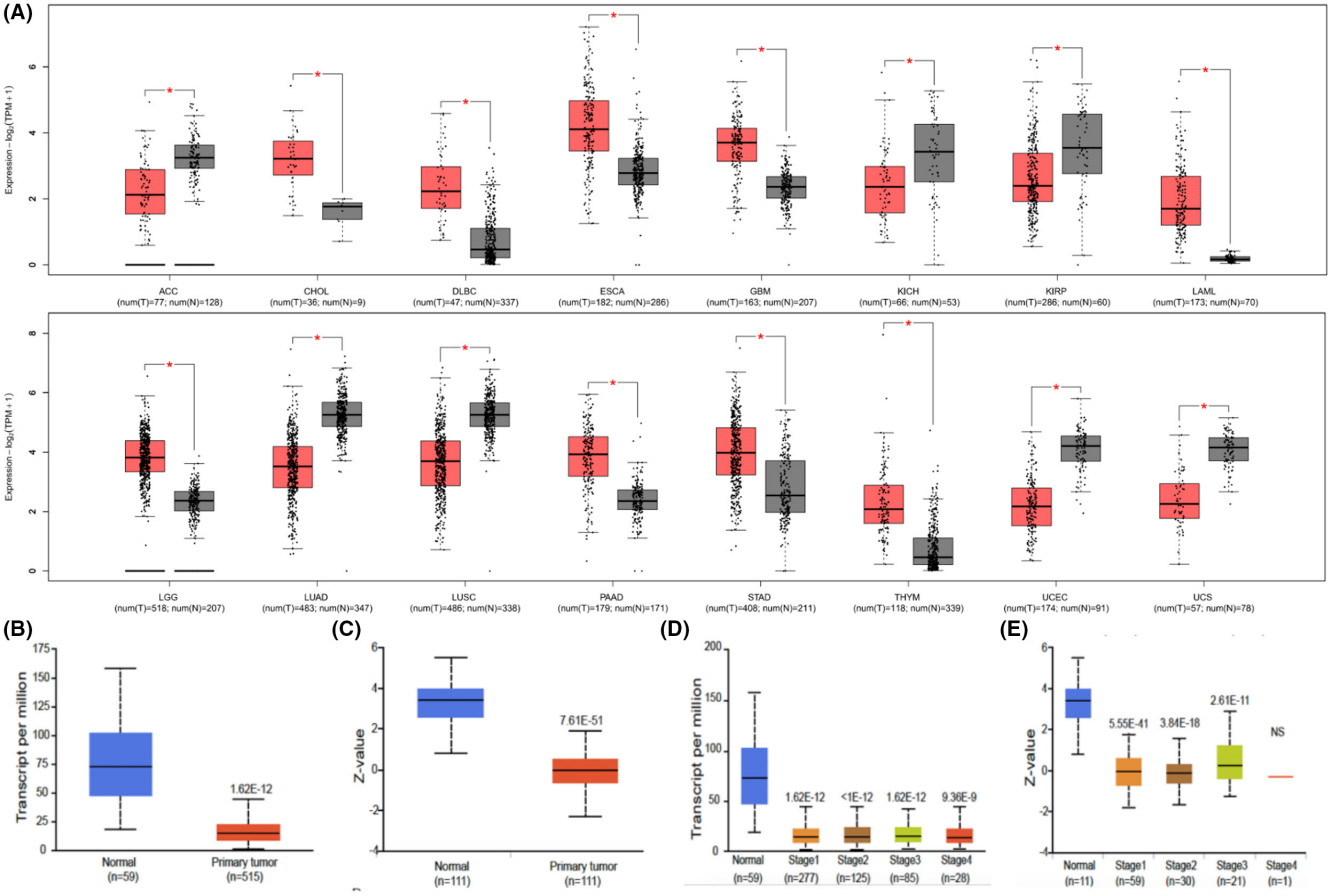
Bioinformatic analysis of HEG1. (A) TCGA and GTEx databases show HEG1 gene mRNA expression differences in cancers and normal tissues. (B, C) UALCAN database shows HEG1 transcription levels in LUAD. (D, E) UALCAN databases show the transcription levels of HEG1 in various tumor stages of LUAD.

In this study, HEG1 expression in a TCGA pan‐cancer dataset and GTEx database were obtained from the GEPIA platform, while UALCAN database was adopted to verify the findings. As the results, HEG1 was dysregulated in LUAD (*p* < 0.001) as shown in Figure 1A–C. We next explored the concentration levels of HEG1 at different TNM stages. Interestingly, HEG1 was reduced in all stages of LUAD, which indicates that HEG1 may play a role in the occurrence and progression of LUAD (Figure 1D and E).

### 3.2 Validation of the expression of HEG1 in clinical LUAD tissues and LUAD cell lines

**FIGURE 2:**
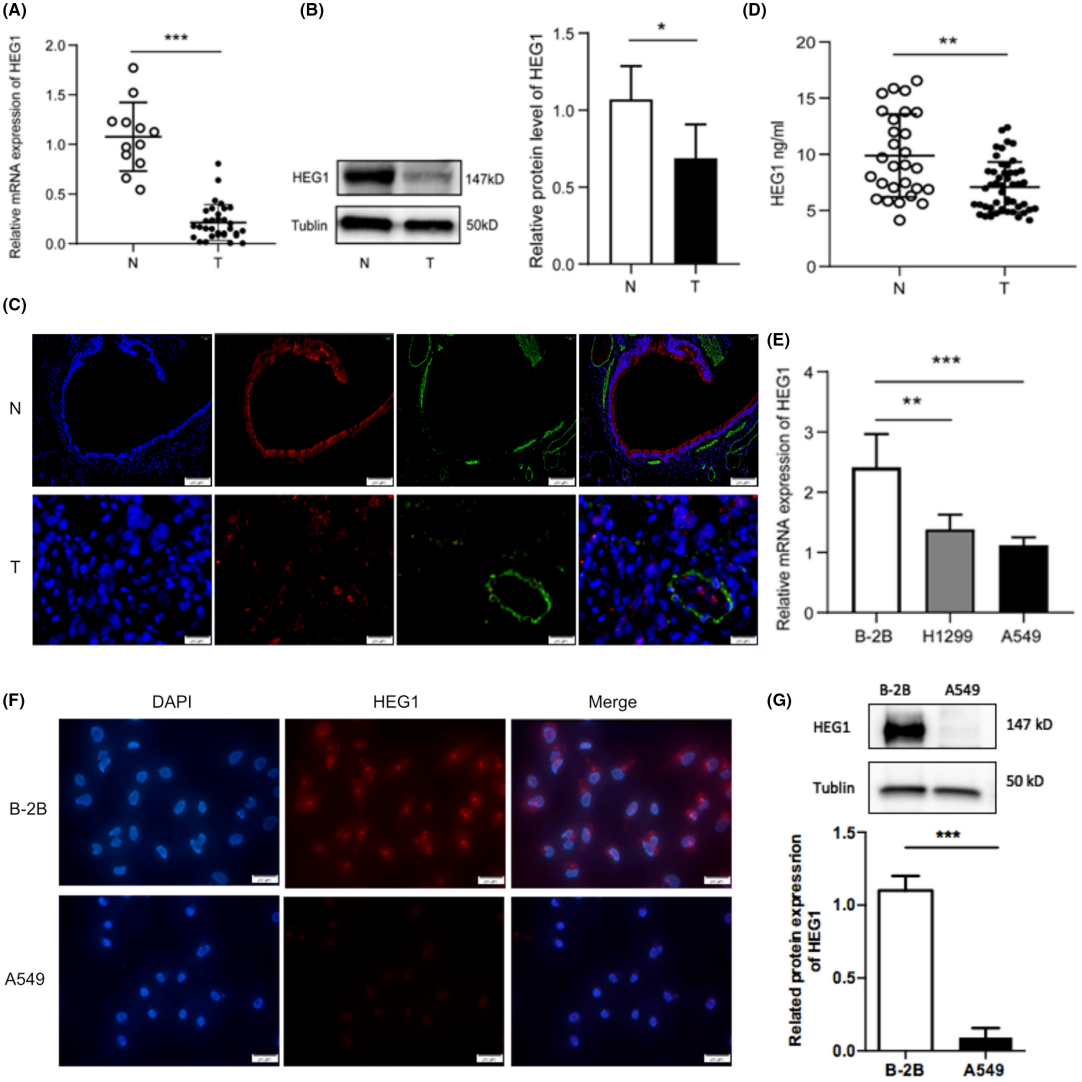
HEG1 was downregulated in lung adenocarcinoma tissues and cell lines. (A) HEG1 mRNA expression levels were determined using qRT‐PCR. (B) The protein levels of HEG1 in LUAD tissues (*n* = 32) and non‐neoplastic lung tissues (*n* = 12) measured by western blot. (C) Immunofluorescence (×200) of tissues from patients with LUAD or BLD (D). The expression level of serum HEG1 in LUAD patients (*n* = 59) and controls (*n* = 28) detected by ELISA. (E) HEG1 expression levels in cell lines BEAS‐2B, A549, and H1299 were determined by qRT‐PCR. (F) Immunofluorescence (×400) and (G) western blot detected HEG1 expression in cell lines BEAS‐2B and A549. Abbreviations: T, LUAD tissues or serum from LUAD patients; N, non‐neoplastic lung tissues or serum from healthy volunteers. *p < 0.05; **p < 0.01; ***p < 0.001.

**FIGURE 3:**
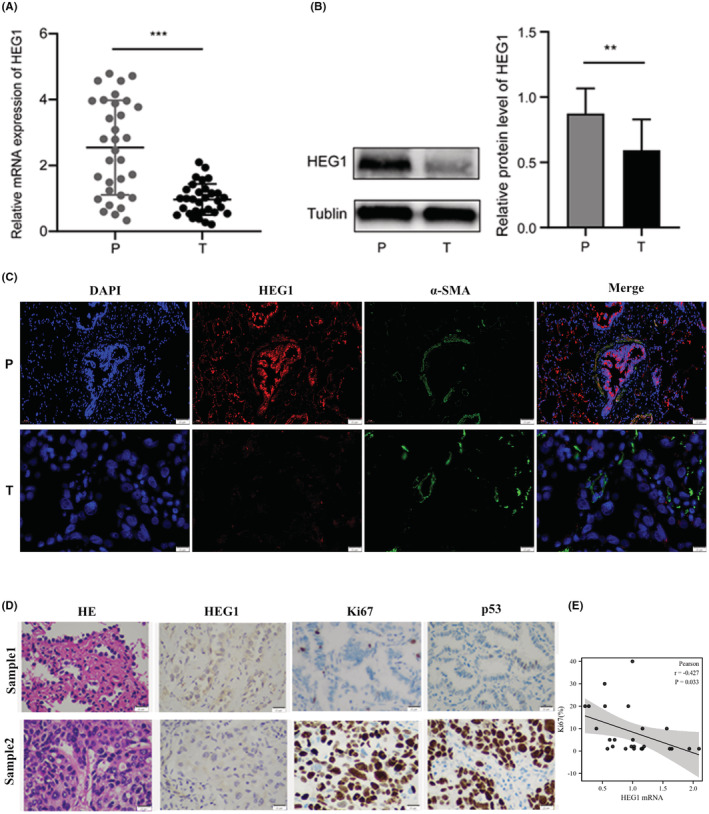
HEG1 was downregulated in lung adenocarcinoma tissues than that in paired adjacent normal tissues. (A) qRT‐PCR determined the mRNA expression levels of HEG1. (B) The protein levels of HEG1 in LUAD tissues (*n* = 32) and paired adjacent normal tissues detected by western blot. (C) Immunofluorescence (×200) measured the protein levels of HEG1 in tissues from one patient with LUAD. (D) H&E staining and the expression of Ki67 and P53 in LUAD tissues detected by immunohistochemistry; (E) Correlation between the patients'Ki67 index and the mRNA expression (*n* = 26). Abbreviations: P, paired adjacent normal tissues; T, lung adenocarcinoma tissues. **p < 0.01; ***p < 0.001.

To validate the results from the TCGA and GTEx databases, HEG1 expression in 32 LUAD tissues and their paired normal lung tissues and 12 benign lung disease (BLD) tissues were detected in mRNA and protein levels, respectively. HEG1 reduced dramatically in LUAD tissues versus normal tissues and BLD tissues at the level of mRNA (*p* < 0.0001, Figures 2A and 3A). Similarly, western blot and immunofluorescence showed that HEG1 decreased in LUAD tissues, as well as the level of mRNA (*p* = 0.044, *p* = 0.007) (Figures 2B,C and 3B,C). Then, we determined the expression level of HEG1 in serum. The results suggested that HEG1 concentration levels decreased in the serum of LUAD patients (*p* = 0.007) (Figure 2D). We further confirmed this result by qRT‐PCR in cell lines BEAS‐2B, A549, and H1299 (Figure 2E). Western blot and immunofluorescence analysis also validated the expression of HEG1 in cell lines. (Figure 2F,G).

Moreover, we found that HEG1 may be related to the degree of tumor differentiation. H&E staining and immunohistochemistry of clinical tissues were used to detect the expression of HEG1, Ki67, and P53 in tumor tissues. As shown in Figure 3D and Figure S1, three cases were diagnosed as poorly differentiated solid adenocarcinoma, other cases were diagnosed as moderately differentiated acinar adenocarcinoma. The p53 protein was mutated in a portion of poorly differentiated tumors in contrast to wild‐type expression in moderately differentiated ones. As expected, poorly differentiated tumors showed lower cytoplasmic HEG1 expression and higher Ki67 staining. Additionally, the Ki67 index was negatively correlated with HEG1 mRNA levels (*r* = −0.427, *p* = 0.033) (Figure 3E). Overall, the findings demonstrated that HEG1 was downregulated in LUAD tissues when compared to paired adjacent normal tissues and that the low HEG1 expression may have a relationship with the degree of tumor malignancy.

### 3.3 HEG1 may act as a potential biomarker in lung adenocarcinoma patients

**FIGURE 4:**
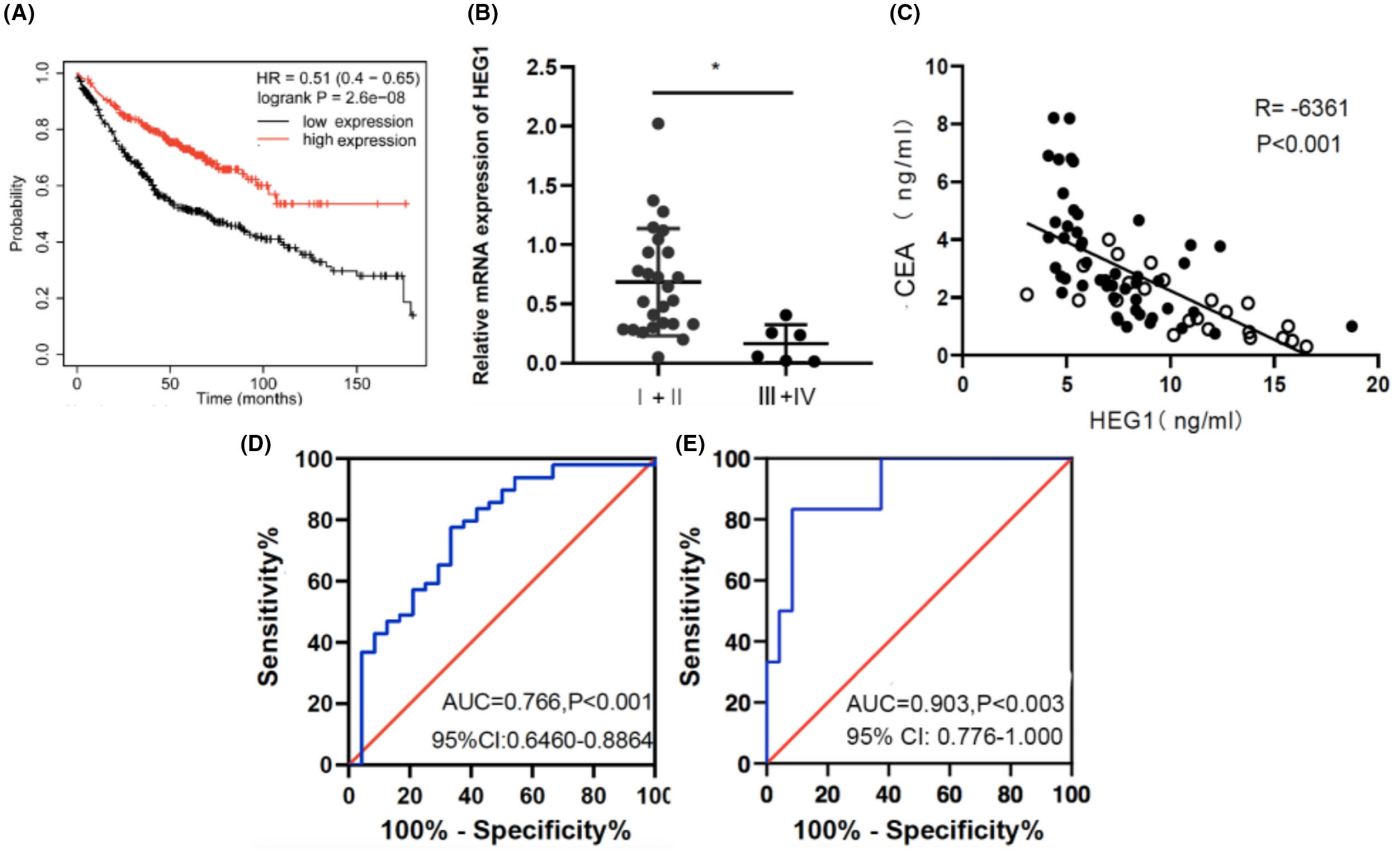
HEG1 may act as a potential serum biomarker for lung adenocarcinoma. (A) Kaplan–Meier Plotter showed the overall survival of LUAD patients with high or low HEG1 expression (Kaplan–Meier Plotter). (B) The mRNA expression levels of HEG1 in different tumor stages of LUAD. Stage I + II (*n* = 26), stage III + IV (*n* = 6). (C) Correlation between HEG1 and CEA in serum of LUAD patients and healthy controls. Solid circles, lung adenocarcinoma patients; Open circles, healthy controls. (D) ROC curves and AUC values for HEG1 in LUAD patients' serum and healthy controls' serum. (E) ROC curves and AUC values for HEG1 in stage I + II and stage III + IV.

In order to assess whether HEG1 is correlated to the clinical pathological parameters of patients with LUAD listed in Table 2, we divided the patients into two groups according to the mean level of HEG1 mRNA. The results showed that patients with an advanced tumor stage had lower HEG1 mRNA expression than the other group (*p* = 0.025) and this manifested that the patients with low HEG1 expression may have a relatively malignant procession. The result of KM plot analysis revealed that patients with high HEG1 expression exhibited longer OS than those patients with low expression (HR = 0.51, 95% CI: 0.40–0.65, *p* = 2.6e‐08) (Figure 4A). We further demonstrated that HEG1 decreased in stage III + IV versus stage I + II (*p* = 0.010) (Figure 4B).

**TABLE 2:** Correlation between HEG1 expression and clinicopathological characteristics in 32 lung adenocarcinoma patients

Variables	No. of case (*n*)	HEG1 high expression rate (%)	HEG1 low expression rate (%)	*p* value
Gender				
Male	18	7(38.9)	11(61.1)	0.821
Female	14	6(42.9)	8(57.1)	
Age (years)				
<65	19	7(36.8)	12(63.2)	0.598
≥65	13	6(46.2)	7(54.8)	
Distant metastasis				
Yes	3	0(0)	3(100.0)	0.132
No	29	13(44.8)	16(55.2)	
Lymph node metastasis				
Yes	9	2(22.2)	7(77.8)	0.185
No	23	11(47.8)	12(52.2)	
TNM stages				
I + II	26	13(50.0)	13(50.0)	0.025*
III + IV	6	0(0)	6(100.0)	

* *p* < 0.05.

Pearson correlation analysis showed that HEG1 was negatively associated with CEA (*r* = −0.636, *p* < 0.001) in serum of LUAD patients and healthy controls (Figure 4C). The area under the ROC curve (AUC) of HEG1 in the diagnosis of LUAD was 0.776 (95%CI: 0.646–0.886, *p* < 0.001) (Figure 4D), with a cut‐off value of 0.464, and the sensitivity and specificity were 77.6% and 66.7%, respectively. ROC curve analysis showed that HEG1 had an AUC of 0.903 (95% CI:0.776–1.000, *p* < 0.001) to distinguish tumor stage with a cut‐off value of 0.257 (Figure 4E), and the sensitivity and specificity were 92.3% and 83.3%, respectively. Overall, these data suggested that HEG1 can be used as a biomarker for LUAD in monitoring tumor recurrence and determining patient prognosis.

### 3.4 HEG1 may affect the progression of LUAD by dysregulation of metabolism

**FIGURE 5:**
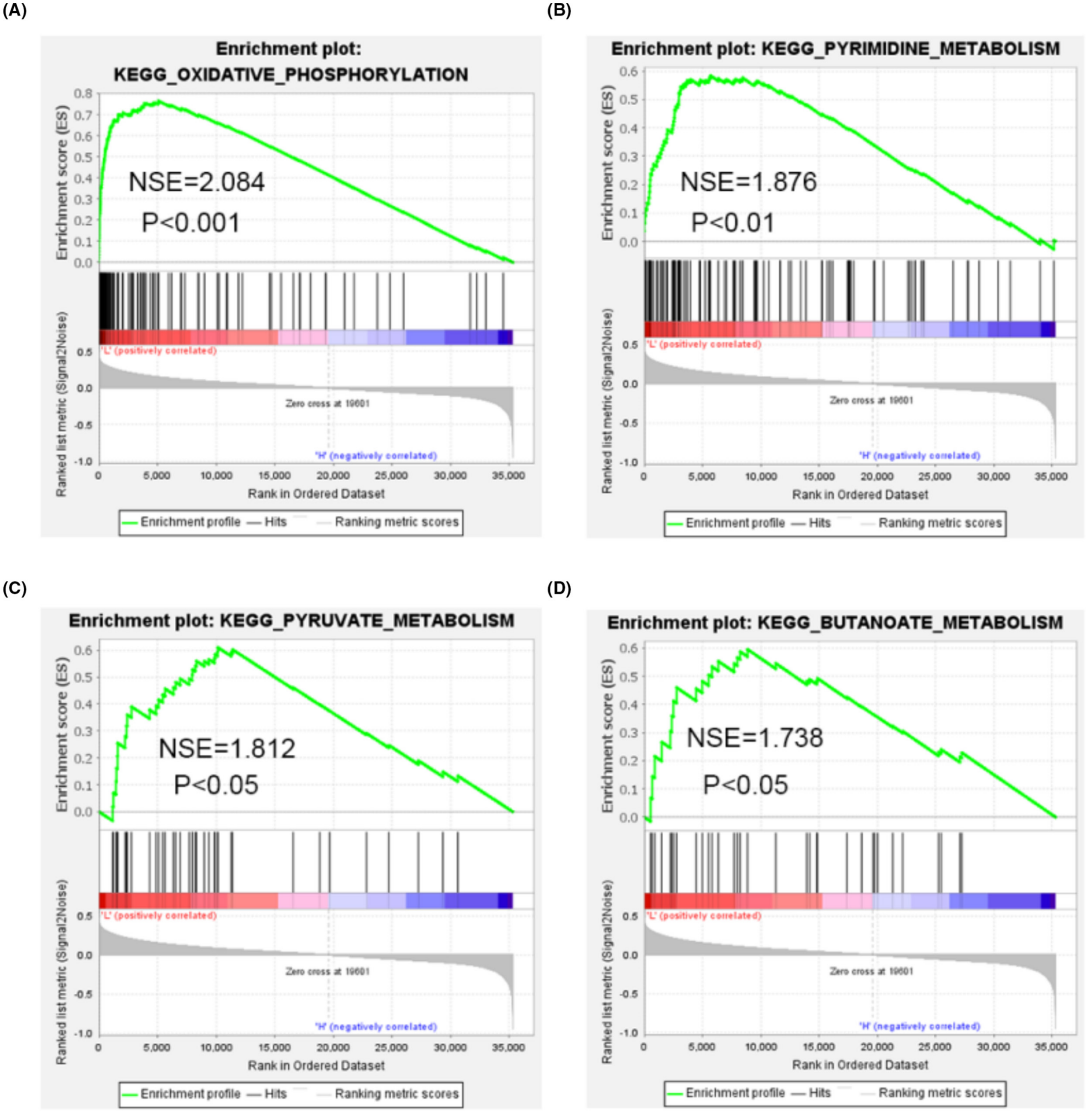
The biological function of HEG1 in LUAD based on GSEA analysis. GSEA results revealed that (A) the Oxidative phosphorylation, (B) Pyrimidine metabolism, (C) Pyruvate metabolism, and (D) Butanoate metabolism were enriched in the low HEG1 expression group. The enrichment scores for each gene are shown in the top panels. The ranking metrics for each gene are displayed on the bottom panels. The metric values are listed on the *Y*‐axis, and the ranks for all genes are listed on the *X*‐axis. NES means normalized enrichment scores.

We conducted GSEA between high and low HEG1 expression datasets to determine the biological functions activated in tumor progression. The enrichment of ‘MSigDB Collection’ indicated significant differences (FDR <0.25, *p* < 0.05) in GSEA, and the specific contents are displayed in Figure 5A,B and Table 3. The findings reveal that when HEG1 expression is reduced, it affects a variety of intracellular signaling networks. ‘Oxidative phosphorylation’ (NES = 2.084, *p* = 0), ‘Pyrimidine metabolism’ (NES = 1.813, *p* = 0.018), ‘Pyruvate metabolism’ (NES = 1.68, *p* = 0.002), and ‘Butanoate metabolism’ (NES = 1.739, *p* = 0.020) are among the metabolic synthesis signaling pathways. Overall, GSEA analysis demonstrated a substantial link between HEG1 and energy metabolism, including glucose metabolism, lipid metabolism, and nucleotide metabolism signaling, implying that these pathways play a role in the LUAD procession of HEG1 (Figure 5A,D).

**TABLE 3:** HEG1 low‐expression related KEGG pathways in lung adenocarcinoma

KEGG pathways	Size	ES	|NES|	NOM *p*‐value
KEGG_OXIDATIVE_PHOSPHORYLATION	113	0.7638673	2.0839224	0
KEGG_PYRIMIDINE_METABOLISM	96	0.5841822	1.8765234	0.0060975607
KEGG_PYRUVATE_METABOLISM	39	0.60998166	1.8129199	0.018367346
KEGG_BUTANOATE_METABOLISM	33	0.59513915	1.7387996	0.020120725
KEGG_NICOTINATE_AND_NICOTINAMIDE_METABOLISM	24	0.5476391	1.7380266	0.003937008
KEGG_GLYOXYLATE_AND_DICARBOXYLATE_METABOLISM	16	0.6481659	1.6917342	0.026

Enrichment Biological Functions from GSEA; KEGG stands for Kyoto Encyclopedia of Genes and Genomes; GSEA stands for Gene Set Enrichment Analysis; ES stands for Enrichment Score; NES stands for Normalized Enrichment Score.

## 4 DISCUSSION

Our study was the first to investigate the expression and clinical significance of HEG1 in lung adenocarcinoma. As the results, HEG1 was decreased in cancer mass and serum from patients with LUAD, while the low HEG1 portends a shorter OS and poorer outcomes for LUAD.

HEG1 is widely distributed^17^ and plays a critical role in the process of myocardial concentric growth,^9^, ^10^ angiogenesis,^18^, ^19^ cell–cell junction^20^ and embryonic development.^11^, ^21^, ^22^ However, there are only few studies on the role of HEG1 in malignant diseases. Zhao et al.^23^ has reported that HEG1 was upregulated and associated with poor prognosis in hepatocellular carcinoma. Recently, HEG1 was observed to be increased and promoting cell proliferation in mesothelioma, HEG1 was considered as a diagnostic biomarker for malignant mesothelioma with specificity and sensitivity reaching 99% and 92%, respectively.^15^ However, inconsistent to mesothelioma and hepatocellular carcinoma, authors also observed negative staining of HEG1 in all 73 LUAD tissues.^15^ Therefore, it is a challenging problem to clarify the role of HEG1 in lung cancer.

To solve this problem, TCGA and GTEx databases were adopted first to compare the expression of HEG1 in cancer mass and normal lung tissue. Unsurprised, HEG1 was dysregulated in LUAD and associated with poor outcome consistent with the previous findings.^15^ To clarify the expression and effect of HEG1 in lung cancer, HEG1 was detected in LUAD tissues and cells by qRT‐PCR, western blot, and immunofluorescence in this study. Consistent with our bioinformatics results, HEG1 was downregulated in both tissue and cells of LUAD. As the expression of HEG1 was regulated by tumor‐suppressor genes and oncogenes concurrently, HEG1 expression could vary in different tumors.^24^ But unlike totally negative staining in Naso JR'study, HEG1 in our study was partially positive stained, which might be contributed by different antibodies adopted in the two studies, for the monoclonal antibody should have higher specificity of than the polyclonal one.^25^, ^26^

HEG1 is only one of the molecules in many complex networks related to cell proliferation,^11^, ^27^ metastasis,^21^ and angiogenesis.^18^, ^19^ HEG1, Ki67, and P53 expression were investigated by H&E staining and immunohistochemistry in the tissues of patients with LUAD. As the results, P53 missense mutation was detected in all cases with low HEG1, whereas the others were p53 wild‐type; HEG1 mRNA were negatively correlated with Ki67 proliferative index in tumor tissues, which suggested that the loss of HEG1 expression led to increased proliferative activity of cancer cells. Overall, HEG1 decreased in LUAD and was associated with higher malignancy. Nevertheless, the mechanisms of HEG1 on LUAD remain unknown.

Next, GSEA analysis was conducted to explore the molecular mechanism of HEG1 on LUAD. HEG1 was associated with cellular metabolism, which implied that HEG1 had potential related pathways with LUAD, as the development and progression of LUAD is linked to a distinct energy metabolism response profile of the micro‐environment, including the metabolism of glucose, lipid, and nucleotides.^28^, ^29^ Currently, we are more inclined to regard HEG1 as a potential solution to our common clinical dilemma.

Due to the lack of an effective and convenient biomarker of prognostic assessment for patients, treatment decisions need to consider anticancer benefits, risks of invasive biopsy, and associated costs.^30^ Our findings indicated that HEG1 might be able to serve as the biomarker in the practical management of patients with LUAD. Reassuringly, the results of serological examinations were consistent with previous bioinformatics analysis and tissues, which provided a basis for our later establishment of a method for HEG1 detection as a biomarker for the prognosis of LUAD patients without invasive biopsy. To verify the reliability of this method, we further investigated the association between the expression of HEG1 and CEA, the typical biomarker for adenocarcinoma.^31^, ^32^, ^33^ Moreover, our results indicated that HEG1 has great clinical significance in the early diagnosis, recurrence monitoring of adenocarcinoma, and has potential to be widely used in clinical practice as a tumor biomarker for diagnosis and prognosis of LUAD.

Nevertheless, our study has several limitations. The prognostic importance of HEG1 needs to be validated in large‐scale studies of LUAD patients in the future. Indeed, the exact pathways affected by HEG1 are still unclear, and further investigations need to be conducted by a series of experiments, including overexpression and knockdown in *vivo* and in *vitro*, in the future. Despite the limitations, this is an instructive and exploratory study to evaluate the link between HEG1 and LUAD with the primary goal of finding a marker for diagnosis.

In conclusion, this study was the first time to investigate the expression and clinical significance of HEG1 in LUAD. HEG1 decreased sharply with the increase in malignancy, and patients with low HEG1 tended to have worse prognosis, advanced stage, and high malignancy, which indicates HEG1 has prognostic significance for LUAD at the clinical level, although further in‐depth study on mechanisms is needed to proceed in the future.

## AUTHOR CONTRIBUTIONS

XZ, YZ, and WH designed the experiments; NW, JS, and QHL analyzed the experimental data.; WMH and WJZ wrote and reviewed the manuscript. All authors approved the final manuscript.

## ETHICS APPROVAL AND CONSENT TO PARTICIPATE

All patients gave their assent to the Institutional Review Board, which allows for a thorough examination of tumor and blood specimens (Ethics committee of the Qingdao Municipal Hospital, Ref.: 2019 LSZ No.109). The registration number of clinical research is ChiCTR2000035343.

## CONFLICT OF INTEREST

There are no competing interests declared by the authors.

## Supporting information


Figure S1

## Data Availability

Data availability statement #10;All data included in this study are available upon request by contact with the corresponding author.
