# Supplementary figures and images for: HEG1 as a novel potential biomarker for the prognosis of lung adenocarcinoma
Source: Cancer Med. 2022 Aug 10;12(3):3288–98. doi: 10.1002/cam4.5081 (PMC9939152; doi:10.1002/cam4.5081)

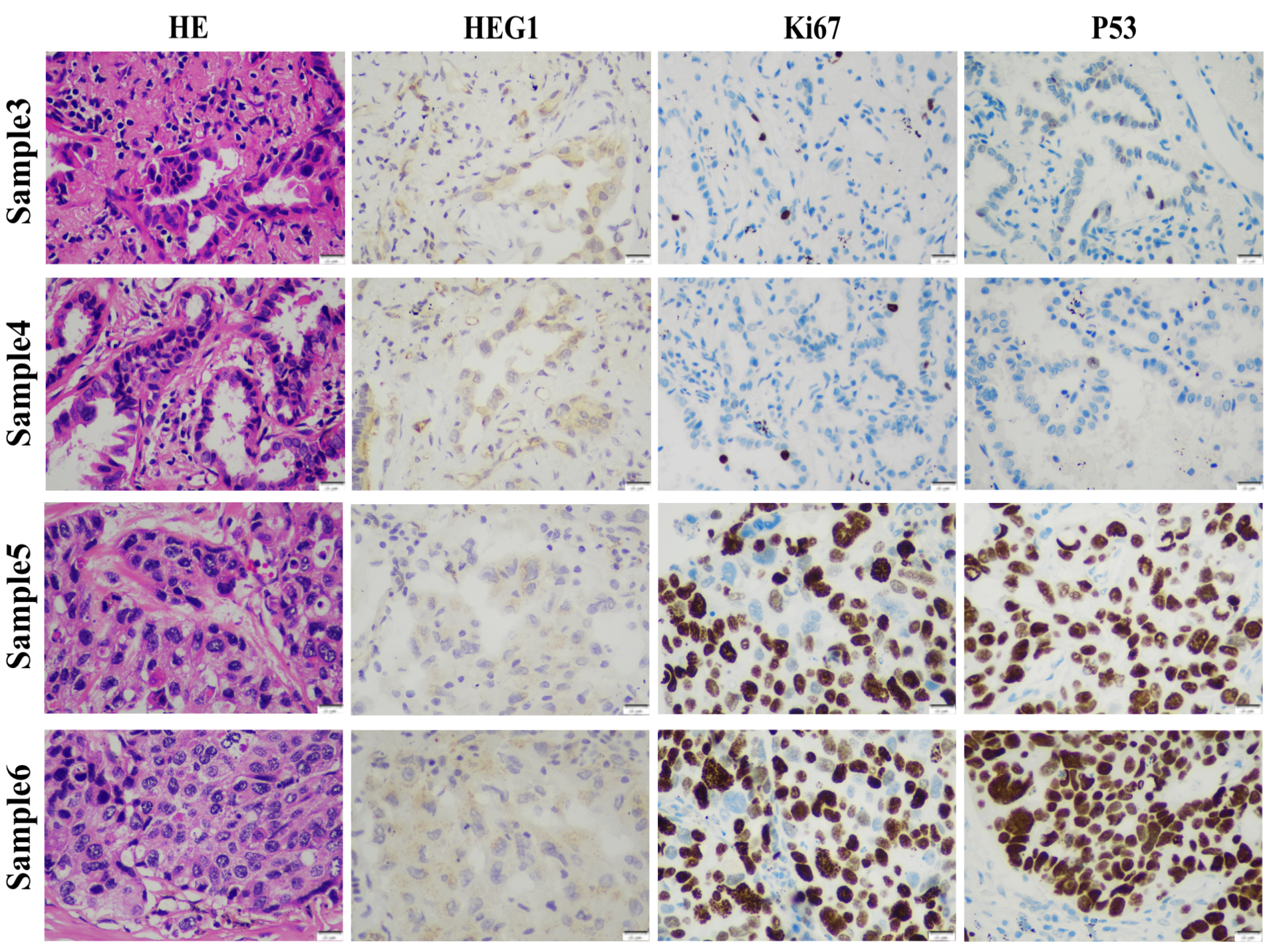

Supplement: Supplementary file 1 — Figure S1 [file CAM4-12-3288-s001.tiff]
